# Monitoring Hearing Function in Patients With Sporadic Vestibular Schwannoma: A Feasibility Study to Inform a Future Randomized Controlled Trial

**DOI:** 10.1097/MAO.0000000000005033

**Published:** 2026-09-11

**Authors:** Daniel Lewis, Rebecca E. Millman, Helen Whiston, Cathal J. Hannan, Andrea Wadeson, Christopher Partlett, Gillian Whitfield, Omar N. Pathmanaban, Kevin J. Munro, On behalf of the STEREO-VS study team

**Affiliations:** a Division of Cancer Sciences, School of Health Sciences, Faculty of Biology, Medicine and Health, University of Manchester, Manchester, UK; b Geoffrey Jefferson Brain Research Centre, Manchester Academic Health Science Centre, Northern Care Alliance NHS Foundation Trust & University of Manchester, Manchester, UK; c Manchester Centre for Audiology and Deafness, Manchester Academic Health Science Centre, University of Manchester, Manchester, UK; d Division of Psychology,Communication and Human Neuroscience, School of Health Sciences, Faculty of Biology, Medicine and Health, University of Manchester, Manchester, UK; e Division of Neuroscience, School of Biological Sciences, Faculty of Biology, Medicine and Health, University of Manchester, Manchester, UK; f Nottingham Clinical Trials Unit, School of Medicine, University of Nottingham, Nottingham, UK; g Clinical Oncology, The Christie NHS Foundation Trust, Manchester, UK; h Division of Cell Matrix Biology and Regenerative Medicine, School of Biological Sciences, Faculty of Biology, Medicine and Health, University of Manchester, Manchester, UK

**Keywords:** Acoustic neuroma, Hearing, Puretone audiometry, Radiosurgery, Speech-in-noise, SRS, Vestibular schwannoma

## Abstract

**Objective:**

There is a need for a definitive RCT comparing upfront stereotactic radiosurgery (SRS) versus watch-and-wait (WW) for long-term hearing preservation in vestibular schwannoma (VS). Specific objectives of this feasibility study were to: (1) assess patient eligibility and recruitment/ retention rates, (2) determine completion rates/ times, and (3) estimate the variance in key proposed outcome measures to inform sample-size estimation for an RCT.

**Study design:**

Nonrandomized, single-centre, prospective study.

**Setting:**

Tertiary-referral centre.

**Patients:**

Thirty-one patients with sporadic VS undergoing WW (n = 22) or SRS (n = 9).

**Intervention(s):**

Four-frequency average pure-tone audiometry (4FAPTA) thresholds, Word Recognition Scores (WRS), monaural/antiphasic Digits-in-Noise speech reception thresholds (DiN SRT_50_), and self-reported outcomes including the Speech, Spatial and Qualities of Hearing Scale (SSQ) and Tinnitus Functional Index (TFI).

**Main outcome measures:**

Feasibility metrics (recruitment, completion rates, testing times). Variance of outcome measures (hearing-related: 4FAPTA, monoaural/ antiphasic DiN-SRT_50_, WRS. Patient-reported outcomes: SSQ, TFI.

**Results:**

Forty-four patients were recruited over 7 months. Thirty-one (70.5%) met inclusion criteria, and 25 (80%) completed all tests. Average time to complete the assessments was 1 hour 42 minutes. Mean 4FAPTA (±SD) in VS-affected ears was 42 ± 14 dB HL (WW) and 55 ± 9.4 dB HL (SRS). Antiphasic DiN-SRT_50 thresholds_ were WW: -14.2 dB SNR ± 4.1; SRS: -11.6 dB SNR ± 1.9, and 4FAPTA and antiphasic DiN-SRT_50_ were correlated (*P* < 0.001) in VS-affected ears.

**Conclusions:**

This study has demonstrated sufficient eligibility and recruitment/retention rates for the proposed RCT and high-test completion rates with acceptable duration. Variability estimates of key outcome measures (DiN-SRT_50,_ 4FAPTA) have also been provided to determine the sample size for the RCT.

## Introduction

Sporadic vestibular schwannomas (VS) are benign tumors arising along the vestibulocochlear cranial nerve^1,2^. They account for 8% of all central nervous system tumors^3,4^, and overall incidence is ~3 to 5.2 per 100,000 person-years, increasing in individuals> 70 years to 20.6 per 100,000 person-years^5^. Treatment options depend on their size, growth rate, and location. Large tumors causing brainstem compression or intractable trigeminal neuralgia usually require microsurgical resection, but for small-to-medium-sized growing tumors, stereotactic radiosurgery (SRS) is an alternative and established therapy^6^. For small VS, an initial “watch-and-wait” approach (WW) with serial MRI is usually adopted, with treatment reserved for tumors showing sustained significant growth^6^.

VS can impact considerably on patient quality-of-life, resulting in auditory and nonauditory symptoms^7–9^. Even without tumor growth, hearing often deteriorates in VS-affected ears, and natural history studies demonstrate that most patients with a conservatively managed VS lose functional hearing at 5 years post diagnosis^9–13^. It has been proposed that SRS delivered at the time of diagnosis may improve long-term hearing outcomes^14–18^, and high-quality systematic reviews have found up to 60% hearing preservation during the first 2 to 5 years following SRS^19–21^. These results are, however, entirely based on single-center case series or matched-cohort studies^18,22^. Other studies of patients on a “watch-and-wait” pathway report similar hearing outcomes to those from high-volume SRS centers^9,23,24^. More evidence is, therefore, needed on the effectiveness of upfront SRS in preserving functional hearing before it can be recommended as a standard of care^25^. Our work with a group of individuals with lived VS experience has identified preservation of hearing function as a priority research question for them. On this basis, we have proposed an RCT of upfront SRS versus WW in the management of VS, to establish the efficacy of SRS in preserving long-term functional hearing.

A key question before such an RCT is how best to monitor hearing function in people undergoing VS treatment. A recent scoping review of hearing outcomes reported following SRS^26^ has highlighted that previous studies used a limited range of hearing-related outcomes, including pure-tone audiometry (PTA), word recognition scores (WRS), and self-reported outcomes. Such measures, however, do not necessarily reflect the real-life hearing difficulties people with VS experience, such as difficulty understanding speech in background noise^26–29^. Within this study, we sought to understand the feasibility of adopting different hearing evaluations within a future planned RCT, including outcome measures such as overall hearing sensitivity (PTA) and measures of the ability to understand speech in noise, such as simulated remote digits-in-noise (DiN) tests^30–32^.

The key aims of this study were to address uncertainties ahead of a planned definitive RCT comparing upfront SRS versus WW for long-term hearing preservation in VS, and specifically to investigate:

1. The proportion of VS patients who would meet eligibility criteria for the RCT along with recruitment and attrition rates.
2. Completion rates and time to complete a proposed outcome measures battery.
3. The within-group (WW, SRS) variance of speech perception in background noise (DiN) and overall hearing sensitivity (PTA), likely co-primary outcomes in a future RCT.

## Methods

### Patient recruitment

Participants with sporadic VS undergoing either WW or SRS were recruited from a single large skull-base neurosurgical center. A flow diagram of patient recruitment is shown in Figure 1. Inclusion criteria included: within 5 years of diagnosis or following treatment with SRS (based on literature that most patients with VS and serviceable hearing undergoing WW or SRS lose functional hearing within the first 5 y^9,10,19,33^); mild-to-severe sensorineural hearing loss in VS-affected ear ( 4-frequency average PTA, 4FAPTA 20-70 dB HL); normal middle ear health; and normal otoscopy results. Participants with NF2-related schwannomatosis, profound hearing loss, tumors with larger than 25 mm extrameatal maximal diameter (who would be unsuitable for radiologic surveillance and the proposed RCT), prior CNS radiotherapy or SRS, and those taking ototoxic medications were excluded. To avoid inclusion of participants with either possible NF2-schwannomatosis or associated age-related hearing loss, individuals younger than 30 years and older than 65 years of age, respectively, were also excluded.

**Figure 1:**
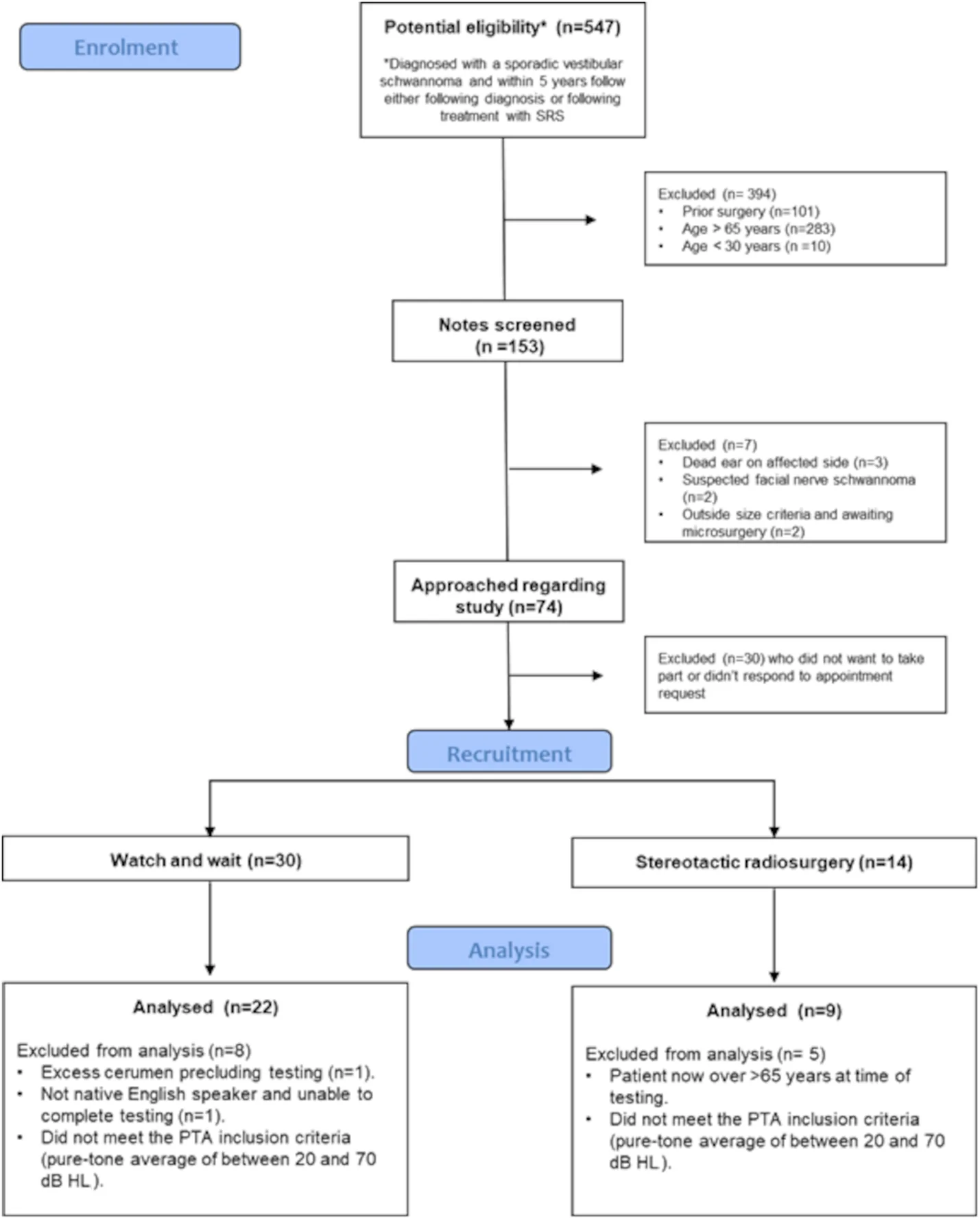
CONSORT diagram detailing number of eligible patients, patients approached, recruitment rates and attrition rates. All patients identified from the institutional database of patients managed with sporadic vestibular schwannoma.

The project received ethical approval from the National Research Ethics Service Greater Manchester East Research Ethics Committee (REC reference 23/NW/0149). Informed consent was obtained from all participants.

### Audiological outcome measures

All testing was performed at a single center. Included hearing outcomes are shown in Table 1, and all participants underwent otoscopy and tympanometry beforehand.

**Table 1:** List of tested audiological outcomes and rationale for inclusion

Hearing Outcome	What Does it Measure?	Reason for Inclusion as an Outcome
Pure-Tone Audiometry	The softest sounds that a person can hear at different frequencies	Gold standard clinical measure of hearing sensitivity and used for diagnosing hearing loss
Word recognition scores	A person’s ability to recognize and repeat monosyllabic words	Standard clinical test of speech perception and used in hearing classification scales
Digits-in-noise	A person’s ability to hear and understand speech in a noisy background	Well-developed method of measuring speech-in-noise perception. Reliable tool for hearing screening and assessing hearing loss; can be delivered in-person or remotely
Tinnitus functional index	Patient-reported outcome of the severity and functional impact of tinnitus on a person’s daily life	Check for symptoms of tinnitus. Can detect a clinically meaningful change in tinnitus severity or impact following treatment or intervention
Speech, spatial and qualities of hearing scale	Patient-reported outcome of hearing disability in everyday listening situations	Assesses 3 broad domains of hearing difficulties, including speech understanding in various listening conditions, how well people can judge the distance or movement of sounds, and the clarity, naturalness, and ease of listening

Pure-tone air-conduction (AC) thresholds for test frequencies of 0.25, 0.5, 1, 2, 3, 4, 6, and 8 kHz were obtained, and the 4FAPTA was defined as the mean AC threshold for 0.5, 1, 2, and 4 kHz. WRS were measured using the Arthur-Boothroyd (AB) word test^34^ for the ear affected by VS only (see supplementary file, Supplemental Digital Content 1, https://links.lww.com/MAO/C532 for further details) and were reported as the mean percentage of correctly repeated phonemes across the test words.

The DiN test assesses correct recognition of digits presented in background noise and uses an adaptive procedure to determine the Speech Reception Threshold (SRT_50_), that is, the signal-to-noise ratio (SNR) in decibels (dB), at which a participant gave a correct response on 50% of the trials.

DiN tests were delivered in a “mock remote setting” (to simulate completion of the test at home in the proposed RCT) using browser-based software. The DiN procedures used have been described previously^35^, and detailed methods are provided in the supplementary file (Supplemental Digital Content 1, https://links.lww.com/MAO/C532). Monaural (ear affected by VS only and contralateral ear only) and “antiphasic” DiN conditions (the phase of the digits is inverted between the ears, while the masker remains the same in both ears; assesses binaural processing ability) were used^32,36^.

### Patient-reported outcome measures

During the same test session, participants were asked to complete both the Speech, Spatial and Qualities (SSQ) Hearing Scale short form^37^ [SSQ-12; a patient-reported outcome measure (PROM) of hearing disability in everyday listening situations], and the Tinnitus Functional Index^38^ (TFI; a PROM designed to measure the severity and functional impact of tinnitus). Further details are provided in the supplementary file (Supplemental Digital Content 1, https://links.lww.com/MAO/C532).

### Data analysis

Clinical feasibility was assessed through eligibility (meeting inclusion criteria), recruitment rates, completion rates, and completion times for the full battery of hearing screening and hearing tests (Table 2).

**Table 2:** Feasibility criteria

Feasibility Test	Go: Proceed Without Modification	Wait: Proceed After Modifications	Stop
Recruitment rate	>80% of target within 12 mo	60%-79% of target within 12 mo	<60% of target within 12 m
Completion rate (%)	>80	60-79	<60
Completion time (h)	<2	>2	>2.5

Hearing-related outcomes were compared across both groups using descriptive measures (means, SDs). Exploratory Pearson correlation analyses (combining data from both groups) of associations between 4FAPTA and DiN-SRTs were carried out. The planned analysis of test-retest reliability of DiN involved the coefficient of variation (CoV), intraclass correlation coefficient (ICC), and visual inspection using the Bland-Altman approach^39^.

## Results

### Clinical feasibility of future RCT

The target recruitment rate was 50-60 participants within 12 months. The “go” criterion for participant recruitment rates was >80% (n = 40) of target within 12 months (Table 2). The recruitment rate “go” criterion was met, as 44/74 participants approached were recruited into the study within 7 months (>6/mo). Of these, n = 22 met the inclusion criteria for the WW group, and n = 9 met the inclusion criteria for the SRS group after testing (Figure 1 describes the reasons why n = 13 participants were excluded after recruitment). No participants withdrew once recruited (0% attrition). Demographics of included participants are presented in Table 3 and the supplementary file (Supplemental Digital Content 1, https://links.lww.com/MAO/C532).

**Table 3:** Participant demographics

		Participant Group			
	WW (N=22)		SRS (N=9)	
	N (%)	Mean (SD)	N (%)	Mean (SD)
Sex				
Male	9 (40.9)		7 (77.8)	
Female	13 (59.1)		2 (22.2)	
Age (y)		55 (5.5)		56 (8.7)
Ethnicity				
White British	20 (90.9)		9 (100)	
Asian/Asian British	1 (4.5)		0 (0)	
Mixed/multiple ethnic groups	1 (4.5)		0 (0)	
Hearing device use				
Bilateral hearing aids	5 (22.7)		0 (0)	
Unilateral hearing aids	8 (36.3)		3 (33.3)	
None	9 (40.9)		6 (66.6)	
Side of VS				
Left	9 (40.9)		5 (55.6)	
Right	13 (59.1)		4 (44.4)	
Koos grade				
I	10 (45.0)		2 (22.2)	
II	7 (31.8)		4 (44.4)	
III	4 (18.2)		1 (11.1)	
IV	1 (4.5)		2 (22.2)	

### Completion rates and time to completion

The “go” criterion of test battery completion within 2 hrs and >80% of tests completed for all participants (Table 2) was met. The average completion time was 1 hour 42 minutes (±15 min, range: 1 h 11 min to 2 h and 20 min). All participants completed PTA, WRS, SSQ, and the TFI, although n = 4 did not report any tinnitus symptoms. Twenty-nine (94%) of the participants who met the PTA inclusion criteria completed the monaural and antiphasic DiN tests (2 DiN data sets were lost due to technical problems). All participants who completed the DiN testing reported that they would be able to perform DiN testing remotely at home with their own earphones and PC/tablet. Only 26% of participants (n = 8) completed the DiN test-retest, due to some participants feeling too tired to attempt the DiN test-retest.

### Estimate the within-group variance of audiological outcome measures (PTA, DiN, WRS)

Table 4 shows the means and variance around the means (SDs) for each hearing outcome measure.

**Table 4:** Hearing outcomes

	Participant Group	
	WW	SRS
	Mean (SD)	Mean (SD)
4FAPTA (dB HL)		
VS ear	42.1 (13.8)	55.1 (9.4)
Contralateral ear	9.7 (6.1)	7.1 (3.4)
Word recognition scores (%)		
Presented at 65 dB SPL to VS ear	71.7 (28.7)	40.9 (27.4)
Adjusted presentation level to VS ear	73.1 (21.7)	47.4 (25.1)
DIN SRT (dB SNR)		
VS ear	-9.9 (1.1)	-10.4 (1.5)
Contralateral ear	-10 (1.7)	-10.9 (1.9)
Antiphasic	-14.2 (4.1)	-11.6 (1.9)
SSQ-12		
Overall	5.5 (2.0)	5.3 (2.1)
Speech	4.9 (2.2)	4.2 (2.4)
Spatial	5.9 (2.7)	5.5 (2.8)
Qualities	5.9 (2.0)	5.9 (1.4)
TFI (%)		
Overall	29.5 (28.3)	13.3 (11.1)
**AAO-HNS class**	**N (%)**	**N (%)**
A	7 (31.8)	0 (0)
B	7 (31.8)	1 (11.1)
C	1 (4.5)	2 (22.2)
D	7 (31.8)	6 (66.7)

The means and variance around the means (SDs) for each hearing outcome measure.

Fourteen patients (64%) in the WW group and only one patient (5%) in the SRS group had serviceable hearing (AAO-HNS class A or B^40^). The 4FAPTA was higher (ie, less sensitive hearing) in the ear affected by VS (WW mean: 42.1dB HL, SD ± 13.8; SRS mean: 55.1dB HL, SD ± 9.4) compared with the contralateral ear (WW mean: 9.7dB HL, SD ± 6.1; SRS mean: 7.1dB HL, SD ± 3.4).

When words were presented at 65 dB sound pressure level (dB SPL) to the VS ear, the WW group (mean: 71.7% correct, SD ± 28.7) performed better than the SRS group (mean: 40.9% correct, SD ± 27.4). After adjustment of the presentation level relative to the average hearing threshold, that is, average AC PTA +25 dB or +30 dB (depending on whether the limits of the audiometer were reached, and to accommodate participants who may have elevated hearing thresholds), the WW group performed similarly to the 65 dB SPL condition (73.1% correct, SD ± 21.7) and the SRS group improved their performance (mean 47.4% correct, SD ± 25.1).

The mean monaural DiN-SRT_50_ in the WW group was -9.9 dB SNR (SD ± 1.1) in the VS ear and –10 dB SNR (SD ± 1.7) in the contralateral ear. In the SRS group, the mean monaural DiN-SRT_50_ was -10.4 dB SNR (SD ± 1.5) in the VS ear and -10.9 dB SNR (SD ± 1.9) in the contralateral ear. In the antiphasic condition, DiN-SRTs were more negative (ie, better performance) for the WW group (mean: -14.2 dB SNR, SD ± 4.1) compared with the SRS group (mean: -11.6 dB SNR, SD ± 1.9).

There was a significant correlation [*r*(29) = 0.67, *P* < 0.001, 2-tailed] between the 4FAPTA in the VS ear and performance on the antiphasic DiN test (Fig. 2A). There were no significant correlations between the 4FAPTA in the contralateral ear and performance on the antiphasic DiN test [*r*(29) = 0.53, *P* = 0.78, 2-tailed; Fig. 2B], or the 4FAPTA in the VS ear and monaural DiN presented to the VS ear [*r*(29) = -0.05, *P* = 0.79, 2-tailed; Fig. 2C]. There was a significant correlation [*r*(29) = 0.46, *P* = 0.012, 2-tailed] between the 4FAPTA in the contralateral ear and performance on the monaural DiN presented to the contralateral ear (Fig. 2D).

**Figure 2:**
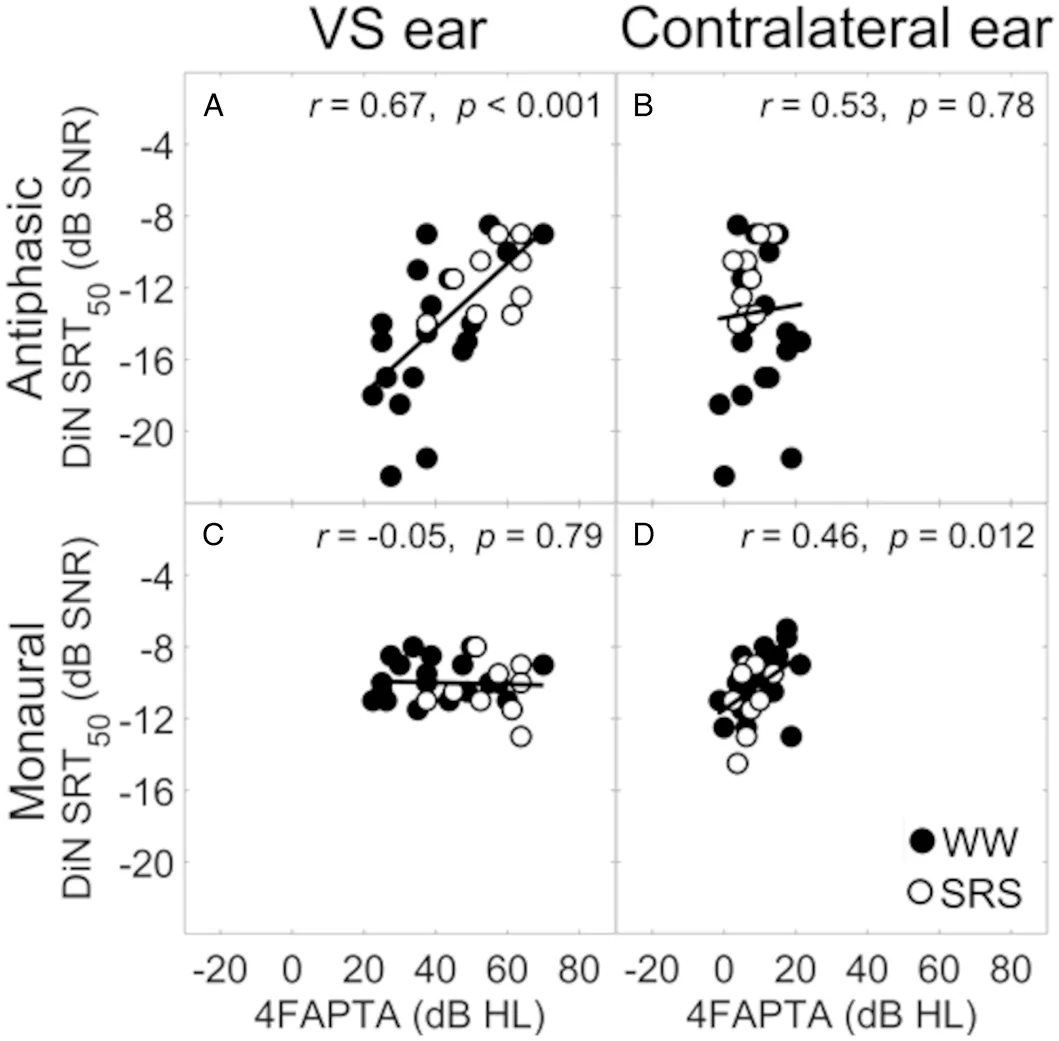
Associations between DiN-SRT_50_ (antiphasic and monaural conditions) and 4FAPTA (in the VS ear and the contralateral ear). Results for the VS ear are shown in the left-hand column, and results for the contralateral ear are shown in the right-hand column. Results for the antiphasic DiN condition are shown in the upper panel (*A and B*), and results for the monaural DiN condition are shown in the lower panel (*C and D*). The WW group is represented by filled symbols, and the SRS group is represented by open symbols. The solid line represents the line of best fit. Correlation coefficients (Pearson *r*) and their associated *P*-values are shown in the top right corner of each subpanel.

As only n = 8 participants repeated the DiN tests in the single test session, no analyses of test-retest reliability were carried out.

#### Self-reported hearing disability (SSQ-12)

Mean overall SSQ-12 scores, where a score of 0 means very poor ability and a score of 10 means very high ability, were similar for the WW (mean: 5.5, SD ± 2) and SRS (mean: 5.3, SD ± 2.1) groups. The mean scores for the 3 subscales were also similar: speech hearing (WW mean: 4.9, SD ± 2.2; SRS mean: 4.2, SD ± 2.4), spatial hearing (WW mean: 5.9, SD ± 2.7; SRS mean: 5.5, SD ± 1.4) and hearing qualities (WW mean: 5.9, SD ± 2; SRS mean: 5.9, SD ± 1.4).

Twenty-seven participants reported symptoms associated with tinnitus. TFI scores were converted to percentages. A TFI score of 0% means no functional impact of tinnitus and a score of 100% means an extreme impact of tinnitus over the past week. The mean TFI score for the WW group (mean: 29.5%, SD ± 28.3) was qualitatively higher (ie, higher impact of tinnitus) than the mean TFI score for the SRS group (mean: 13.3%, SD ± 11.1).

## Discussion

Within this prospective study we have demonstrated clinical feasibility (eligibility, recruitment, and attrition rates; test completion rates and testing time) of undertaking a future definitive RCT of upfront SRS versus WW for long-term hearing preservation in VS. We have furthermore obtained estimates of the variances in key outcome measures identified as being most important to people with VS, supporting feasibility of obtaining a sufficient sample size recruitment for such an RCT.

### Informing the future RCT

Since we completed the feasibility testing, we have carried out a dedicated series of PPI workshops with a group of individuals with lived VS experience to refine the list of hearing outcomes to be used in the RCT. This PPI group clearly identified that the main concerns for people living with VS are that hearing loss prevents them from detecting some sounds and/or understanding speech in background noise. Hearing deterioration over 4 years, measured by (1) detection of sounds using PTA, and (2) speech-in-noise (DiN) recognition, was agreed with the PPI group as co-primary outcomes for a future RCT. Within this RCT, our proposal is to measure DiN remotely, and a key finding of this study is that all participants who completed the DiN testing reported they would be able to complete remote DiN testing at home.

Within the future RCT and based on input from our PPI group members, who highlighted the importance of regularly monitoring changes in hearing, hearing-related outcomes will be measured at baseline following entry into the trial and then at 6, 18, 30, and 48 months. We have chosen a total follow-up duration of 4 years based on evidence showing rapid hearing deterioration (in both untreated VS and post-SRS tumors) that slows by 4 to 5 years post diagnosis^19,40^, with a substantial proportion of patients having measurable hearing deterioration^10,41^ by the fourth year. A total follow-up period of 4 years will also allow for more complete resolution of any posttreatment tumor swelling, which usually resolves within 2 years and could be associated with changing symptoms^42^.

The results of this study furthermore provide variance estimates for each co-primary outcome informing sample size calculation. Our PPI group indicated that the minimally clinically-important difference (MCID) for each co-primary outcome would be a 10 dB PTA difference and a 2 dB DiN-SRT_50_ difference (representing a reduction in speech recognition performance of ~10%) at 5 years. For a marginal power of ≥90% and allowing for a 20% loss to follow-up, a total target sample size of 216 (108 per group) for the proposed future RCT was estimated. Across the UK, there are >1500 newly diagnosed VS patients annually, with ~ 50% of these patients being potentially eligible for a future RCT based on size and hearing criteria^43^. Within this 7-month feasibility study, we recruited 44 patients (>6 patients/mo), demonstrating that a monthly randomization rate of > 0.5 patients/center for a multicentre RCT is feasible.

### Evaluation of proposed hearing outcomes

#### Pure-tone audiometry

Most patients with a conservatively managed VS and serviceable class A or B hearing^33^ at presentation lose serviceable hearing within the first 5 years post diagnosis, with the rate of hearing decline then slowing^9–13^. Within this feasibility study, the mean follow-up duration after diagnosis was 2.24 years (range: 0.28 to 4.88 y) for the WW cohort, with 64% of WW patients having serviceable class A/B hearing at this point (a percentage in keeping with published literature)^9–13^.

Like patients undergoing WW, following SRS, high rates of hearing preservation in the initial 2 to 3 years following treatment are seen, followed by a significant deterioration up to 5 years post diagnosis^19,44,45^. Compared with the WW cohort, the previously SRS-treated cohort had comparably worse hearing measures, with only 1/9 patients in this cohort having serviceable class A/B hearing. Comparing hearing outcomes in this nonmatched cohort was not a specific aim of this study. Although the average time between SRS treatment and audiological assessment was comparable at 1.99 years, unlike the WW cohort, all tumors in the SRS group had a prior history of significant growth necessitating treatment. This is reflected in the larger size of this cohort and, given the reported relationship between tumor growth and hearing loss in the literature^46^, could in part explain the worsened hearing in this group.

#### Digits in noise testing

To our knowledge, this is the first study to use both antiphasic^32,36^ and monaural^47^ DiN testing in participants with VS^26^. Compared with monaural testing, which is not representative of real-life listening scenarios, the antiphasic DiN test is more sensitive to the effects of unilateral sensorineural hearing loss and the associated binaural antiphasic processing disadvantage^32^. In prior studies of hearing-impaired individuals, monaural DiN-SRTs correlated strongly with PTA^47^. Whereas a significant correlation between the PTA results in the VS ear and performance on the antiphasic DiN test was observed in our study, no significant correlations between the PTA in the VS ear and monaural DiN were observed. The reason for the differing association between the antiphasic and monaural DiN test results in the affected ear and measured PTA is unclear. Participants self-calibrated the DiN presentation level to ensure digits were audible. Participants with higher PTA thresholds may have increased the DiN presentation level such that cross-hearing by the contralateral cochlea was possible. This cross-hearing may result in a better DiN performance than expected in the VS ear, further supporting use of antiphasic DiN rather than monaural DiN testing for monitoring asymmetric hearing loss in participants with VS.

Overall, our results indicate that PTA results do not fully capture the variance in speech-in-noise results and underestimate the hearing difficulties experienced by people with VS, and provide further justification for inclusion of DiN testing as a co-primary outcome within the proposed RCT. Previous studies comparing PTA, WRS and a QuickSIN test in patients with VS and control individuals have demonstrated that QuickSIN asymmetry was more predictive of VS-affected ears than asymmetry in either PTA or word-recognition-in-quiet measures^48^.

Although we could not evaluate the test-retest reliability of DiN, previous work suggests that the test–retest differences for DiN are low regardless of the hearing status of participants (normal-hearing or listeners with hearing loss^49,50^) or the test environment (both laboratory-based and home-based testing^30,49^), with one study of 28 ears, for example, showing a test-retest variability of only 0.7 dB^49^.

### Patient-reported outcome measures

To our knowledge, no previous studies have used the full SSQ Hearing Scale^51^ or the short form SSQ-12^37^ to measure hearing disability following SRS treatment (specifically) for VS. Our results indicate that the mean overall SSQ-12 scores were similar for the WW and SRS groups, with similar scores for the speech hearing, spatial hearing, and hearing qualities subdomains. Prior studies^52,53^ have administered the SSQ-12 to participants who underwent microsurgery for VS, demonstrating both longitudinal changes in SSQ-12 score posttreatment and significantly greater hearing disability, in comparison with control participants matched on age, sex, and hearing level.

In line with our PPI group responses and choice of co-primary outcome measures, the greatest levels of hearing disability (SSQ scores of ~2 to 4.5) were associated with understanding speech in the presence of noise, listening to multiple streams of speech and switching (eg listening to someone speaking and the television at the same time), localizing sounds and listening effort.

The pretreatment prevalence of tinnitus in patients with VS is estimated to be around 70%^54^, and reducing tinnitus symptoms is a patient-preferred outcome for about one-third of patients with VS^28^. Tinnitus symptoms may worsen in patients who are managed conservatively^55^, and knowledge about tinnitus outcomes could influence management for these patients^56^. Here we used the TFI to characterize tinnitus severity and the impact of tinnitus on the daily lives of our participants, and the TFI may be more responsive to treatment-related changes over time^54,57^.

Recent systematic reviews^56,58^ have identified a complex relationship between different VS treatments (microsurgery, SRS) and tinnitus outcomes. Prior studies are highly heterogeneous, with some demonstrating an overall decrease in tinnitus burden post-SRS^59^, whereas others have shown worsened self-reported measures of tinnitus following gamma-knife radiosurgery^60^. King et al.^58^ noted that future studies should include a conservatively managed WW group to characterize the natural progression of tinnitus in people with VS and help refine treatment strategies. Indeed, the lack of high-quality data on this subject has informed our decision to include robust patient self-reported measures of tinnitus severity within the outcome panel for the planned RCT.

### Limitations

To our knowledge, ours is the first study to compare antiphasic and monaural DiN, PTA, and patient self-reported QOL measures in a VS population, and to provide an estimate of the feasibility of undertaking this outcome measures panel within an RCT. There were, however, key differences between this single-center feasibility study and the proposed future multicenter RCT that should be considered.

The proposed RCT will recruit newly-diagnosed participants with serviceable hearing at presentation. Although this feasibility study included individuals within 5 years of diagnosis/treatment, the within-group variance of hearing outcome measures identified may not fully extrapolate to the newly-diagnosed VS population. Individuals >65 yrs of age were also excluded. These exclusion criteria will not be used within the proposed RCT, and the estimated feasible recruitment rates from a large skull base-center for this RCT may, therefore, be an underestimate. Second, except for one patient who had been treated at a different center, all SRS-treated patients underwent LINAC-based therapy. Studies have suggested that a mean cochlear dose of <4 Gy is associated with improved hearing outcomes^61,62^. There is limited high-quality evidence, however, on the effectiveness of one modality over another with regard to long-term hearing preservation^25^, and within the planned RCT, we have proposed a pragmatic trial design with the choice of SRS platform being determined by best usual practice at each participating site.

Finally, although we evaluated SSQ and TFI within this feasibility study, people with VS report several other nonauditory symptoms^7–9^ not fully evaluated in this study, including anxiety, headaches, dizziness, fatigue, and impairment of cognitive function and memory. Based on a recent core outcome set^63^  for asymmetric hearing loss and domains identified as priorities by our PPI group, within the proposed RCT we will be including, alongside SSQ, other PROMs that capture these symptoms such as the Revised Hearing Handicap Inventory—Screening^64^, a VS—specific quality-of-life measure (Penn Acoustic Neuroma Quality of Life^65^) and the Generalized Anxiety Disorder-7^66^. The Theoretical Framework of Acceptability (TFA)^67^ will also be included to assess the acceptability of any new health care interventions within the trial and ensure that patient perspectives guide the interpretation and implementation of trial results.

## Conclusions

This study has demonstrated the clinical feasibility of undertaking a proposed future definitive RCT comparing upfront SRS versus WW for long-term hearing preservation in VS, using primary outcome measures that are important to patients. Variability estimates of patient-driven co-primary outcome measures have been provided, and these furthermore support the feasibility of obtaining a sufficient sample size for such an RCT.

## Sources of funding

The study was part-funded by the NIHR Manchester Biomedical Research Centre (NIHR203308).

## Supplementary Material
