# Supplementary material for: Monitoring Hearing Function in Patients With Sporadic Vestibular Schwannoma: A Feasibility Study to Inform a Future Randomized Controlled Trial
Source: Otol Neurotol. 2026 Sep 11;47(10):e1217–25. doi: 10.1097/MAO.0000000000005033 (PMC13747291; doi:10.1097/MAO.0000000000005033)
Supplement: Supplementary file 1 [file mao-47-e1217-s001.docx]

## Supplementary Methods:

### Pure-tone audiometry

PTA was performed in a double-walled sound-attenuating booth. Pure-tone air-conduction (AC) thresholds for test frequencies of 0.25, 0.5, 1, 2, 3, 4, 6, and 8 kHz were obtained from both ears using British Society of Audiology (2018) recommended procedures. A GSI Pello audiometer and DD450 supra-aural headphones were used for PTA. The 4-frequency average PTA (4FAPTA) was defined as the arithmetic mean of the air-conduction thresholds for test frequencies of 0.5, 1, 2 and 4 kHz.

### Word recognition scores

WRS were measured using the Arthur-Boothroyd (AB) word test^1^ for the ear affected by VS only. AB words, which all consist of three phonemes, were presented using a GSI Pello audiometer and DD450 headphones at two different presentation levels: 65 dB SPL and the average AC PTA + 25-30 dB (depending on whether the limits of the audiometer were reached, and to accommodate for this population who may have elevated hearing thresholds). Speech-shaped noise was presented to the contralateral ear during testing to prevent cross-hearing. The masking calculation for the speech noise level was based on formula 2b from Coles and Priede^2^:

Masking level = D_s_ + E_m_ + max ABG_nt_ – 40	(1)

Where D_s_ is the setting of the speech intensity dial on the audiometer and E_m_ is the difference between in the audiometer dial readings for speech and noise “*at which the intelligibility of speech, presented to normal-hearing listeners at a level giving >95% correct response without the noise, is reduced to 10% when mixed with the noise in the same earphone*”^2^. The max ABG_nt_ is the maximum air-bone gap in the contralateral ear at any one frequency in the range of 0.25-4 kHz and 40 dB is the minimal interaural attenuation for the headphones.

Each participant completed three AB word lists of ten words (30 words in total) for both WRS stimulus presentation levels. WRS were reported as the mean percentage of correctly repeated words and phonemes for the word lists delivered at each presentation level.

**Digits-in-Noise testing**

DiN tests were delivered in a “mock remote setting” using browser-based software within the “Manchester Online Speech-Perception Suite.” The procedures and stimuli used in the present study have been described previously^3^. Before testing began, the DiN software provided plain language test instructions, a demonstration, and a subjective calibration stage that allowed participants to determine the presentation level of the stimuli^3^). DiN testing was preceded by an unscored practice block containing only two turns. A subset of participants were also asked to repeat the DiN tests to measure its test-retest reliability in participants with VS.

Participants were seated in front of a computer and monitor in a double-walled sound-treated room. The stimuli were presented over Sennheiser HD 300 headphones connected to a HP EliteBook 640 laptop. Participants were asked to enter responses using a mouse and an on-screen number pad. As our participants had hearing loss in at least one ear, the stimulus level was determined by the participant in a subjective calibration stage. This stage ensured that all stimuli were both audible and comfortable for the participants. Participants were asked to listen to a carrier phrase “The digits …” followed by a random sequence of three digits (e.g. “5 … 6 … 2”) sampled without replacement from the range 1–9. The carrier phrase and the digits were presented in a background of speech-spectrum-shaped Gaussian masking noise of the same bandwidth as the target speech The carrier phrase, digits and background noise were presented under monaural (ear affected by VS only and contralateral ear only) and “antiphasic” (the phase of the digits is inverted between the ears, while the masker remains the same in both ears) conditions, as the antiphasic DiN test may be more sensitive to asymmetrical hearing losses than monaural or diotic (i.e. the same stimulus presented to both ears) DiN. The order of these test conditions was fixed as 1) practice, 2) antiphasic, 3) right ear, 4) left ear for each participant.

Correct entry of at least two of three digits was considered as a correct response for each trial. Visual feedback concerning task difficulty and correct responses was provided to participants following each trial. The SNR started at +2 dB for each adaptive track and then varied following a two-down, one-up tracking rule. For the first two turn points, the SNR varied in 6 dB steps. For the last four turn points, the step size was 2 dB. The SRT_50_ was defined as the mean of the SNRs at the final four turn points. The final DiN result for each participant is expressed as the SRT_50_ (in dB SNR), where lower (more negative) values indicate better ability to understand speech in noise.

**Patient reported outcome measures**

The Speech, Spatial and Qualities of Hearing Scale (SSQ) short form^4^ is a PROM of hearing disability in everyday listening situation. Both the long^5^ and the short form SSQ^4^ assess 3 broad domains of hearing: 1) Speech Hearing measures speech understanding in various listening conditions (e.g. “You are in a group with several people talking. Can you follow the conversation?”), 2) Spatial Hearing measures how well people can locate and judge the distance or movement of sounds (e.g. “Can you tell how far away a bus or a truck is, from the sound?”), and 3) Qualities of Hearing measures the clarity, naturalness and ease of listening (“Do you have to concentrate very much when listening to someone or something?”). The SSQ-12 contains 12 items, with 4 items per broad domain of hearing. Each item is rated on a scale of 0-10 where a score of 0 means “not at all” i.e. very poor ability and a score of 10 means “extremely well” i.e. very high ability. The SSQ-12 score for each domain is the mean of the responses for a given domain. The total SSQ-12 score is the mean of the responses to all 12 items.

The Tinnitus Functional Index (TFI^6^) is a PROM designed to measure the severity and functional impact of tinnitus. The TFI contains 25 items representing 8 domains: Intrusiveness, Sense of Control, Concentration, Sleep, Auditory, Relaxation, Quality of Life and Emotional. Respondents are asked to provide a score on a scale from 0-10 where a score of 0 means “not at all” and a score of 10 means “extremely” in terms of how much impact their tinnitus has had over the past week. The scores for each item within a domain can be averaged. The overall TFI score is calculated by the mean of the responses to all 25 items and then multiplying this by 10 to give a total score between 0 and 100. The TFI can be administered pre- and post-treatment to detect a clinically meaningful change (≥13 points in the total TFI score) following treatment or intervention.

## Supplementary Results:

### Patient demographics

There were more females (59.1 %) than males (40.9%) in the WW group but more males (77.8%) than females (22.2%) in the SRS group. The mean age of participants in the WW group was 55 years (+/- 5.5 yrs, range 45-64 yrs) and the mean age of participants in the SRS group was 56 years (+/- 8.7, range 34-63 yrs). Most of the participants identified as White British (90.9% of WW, 100% of SRS).

A small number of participants reported that they use bilateral hearing aids (22.7% in WW group, 0% in SRS group). About one third of each group reported that they use a hearing aid or Contralateral Routing of Signal aid in one ear only (36.3% in WW group, 33.3% in SRS group). Most of the participants in both groups reported that they do not use hearing aids (40.9% in WW, 66.6% in SRS group).

For the WW group (radiological surveillance), mean follow-up duration after diagnosis was 2.24 years (range 0.28 – 4.88 years), and except for one tumour that showed serial growth, all tumours had remained stable or static on serial MRI follow-up. Ten tumours (45%) were purely intracanalicular Koos grade I tumours, seven tumours were Koos grade II, and five tumours were Koos grade 3 or 4. For the SRS treated cohort, the mean time between SRS treatment and audiological assessment was 1.99 years (range 0.60 – 3.27 yrs). All participants had a history of clinically significant tumour growth prior to SRS treatment, with 8/9 participants in the SRS group having undergone Linac based SRS and one patient having been previously treated with the gamma-knife system. At time of SRS treatment, two tumours were intracanalicular, four tumours were Koos grade II, and three tumours were Koos grade 3 or 4. At time of audiological assessment, eight tumours were stable or shrinking, with one tumour displaying serial growth post gamma-knife radiosurgery.

## References:

1	Boothroyd A. ‘developments in speech audiometry’. *Int J Audiol* 1968; **7**: 368.

2	Coles RR, Priede VM. Masking of the non-test ear in speech audiometry. *J Laryngol Otol* 1975; **89**: 217–226.

3	Shehabi A, Plack CJ, Prendergast G, Munro KJ, Stone MA, Laycock J *et al.* Online Arabic and English Digits-in-Noise Tests: Effects of Test Language and At-Home Testing. *J Speech Lang Hear Res* 2025; **68**: 388–398.

4	Noble W, Jensen NS, Naylor G, Bhullar N, Akeroyd MA. A short form of the Speech, Spatial and Qualities of Hearing scale suitable for clinical use: the SSQ12. *Int J Audiol* 2013; **52**: 409–412.

5	Gatehouse S, Noble W. The Speech, Spatial and Qualities of Hearing Scale (SSQ). *Int J Audiol* 2004; **43**: 85–99.

6	Meikle MB, Henry JA, Griest SE, Stewart BJ, Abrams HB, McArdle R *et al.* The tinnitus functional index: development of a new clinical measure for chronic, intrusive tinnitus. *Ear Hear* 2012; **33**: 153–176.
